# Digital and technology‑enabled approaches for post‑stroke fatigue: A scoping review protocol

**DOI:** 10.1371/journal.pone.0352714

**Published:** 2026-10-29

**Authors:** Xiangdong Huang, Jiangmei Xu, Lingmin Wang, Shiting Huang, Xiaoping Zhu

**Affiliations:** 1 Nursing Department, Shanghai Tenth People's Hospital, School of Medicine, Tongji University, Shanghai, China; 2 Emergency Department, Shanghai Tenth People's Hospital, School of Medicine, Tongji University, Shanghai, China; Federal University of Paraiba, BRAZIL

## Abstract

**Background:**

Post‑stroke fatigue (PSF) affects approximately 50% of stroke survivors and is one of the most common and disabling post‑stroke sequelae. Digital and technology‑enabled approaches have attracted increasing attention in the field of stroke rehabilitation, including mobile health applications (mHealth), wearable devices, virtual reality (VR), remote rehabilitation, artificial intelligence (AI), and neuromodulation technologies. However, no comprehensive scoping review has systematically mapped the full spectrum of evidence on digital and technology‑enabled approaches for PSF.

**Methods:**

This scoping review will follow the Joanna Briggs Institute (JBI) methodology for scoping reviews, be guided by the Population, Concept, Context (PCC) framework, and be reported according to the Preferred Reporting Items for Systematic Review and Meta‑Analysis Protocols (PRISMA‑P) and the Preferred Reporting Items for Systematic reviews and Meta‑Analyses extension for Scoping Reviews (PRISMA‑ScR). Eight electronic databases (PubMed, Embase, Web of Science, CINAHL, Cochrane CENTRAL, IEEE Xplore, ACM Digital Library, and PsycINFO) and five gray literature sources (OpenGrey, ProQuest Dissertations and Theses Global, ClinicalTrials.gov, the World Health Organization International Clinical Trials Registry Platform, and Google Scholar) will be searched. Two reviewers will use NoteExpress to remove duplicates and manage references, and will independently screen titles, abstracts, and full texts. After pilot testing, data will be extracted independently by the two reviewers. Synthesis will involve descriptive numerical summaries and inductive qualitative content analysis. Inter‑rater agreement will be assessed during a calibration phase. The expected output is an evidence map organized by approach category and purpose (assessment, monitoring, prevention, and treatment), which will identify knowledge gaps and guide future research.

**Registration:**

This protocol has been registered on the Open Science Framework (OSF). Registration DOI (https://doi.org/10.17605/OSF.IO/4WMEK).

## 1. Introduction

Stroke is the second leading cause of death globally and the third leading cause of combined death and disability, with a lifetime risk of approximately 25% for adults aged 25 years and older [1,2]. As acute stroke care continues to improve, a growing number of survivors live with long‑term sequelae [3]. Among these, post‑stroke fatigue (PSF) is one of the most common sequelae, affecting about 50% of stroke survivors regardless of stroke type or time since onset [4,5]. Recent cohort studies report an incidence of PSF as high as 40%–60% in the acute phase [6]. Despite its high prevalence, PSF remains under‑recognized and poorly managed in routine stroke care; consequently, clinicians have limited evidence‑based management options [7].

The impact of PSF spans multiple domains of function and participation. For example, stroke survivors describe PSF as an overwhelming sense of physical and mental energy depletion that severely affects quality of life, impedes return to work, limits social participation, and reduces engagement in rehabilitation [8,9]. Multi‑country surveys indicate that health professionals lack evidence‑based guidance for managing PSF, and digital approaches are rarely considered in current clinical pathways [10]. Real‑time measurement studies show that PSF fluctuates throughout the day, and retrospective scales cannot adequately capture its dynamic nature — highlighting the need for continuous digital monitoring methods [11]. A recent exploratory study on patient experience found that post‑stroke cognitive fatigue is perceived as a dimension distinct from physical fatigue, with implications for the design of targeted digital approaches [12]. Patients and caregivers consistently rank fatigue as a priority outcome, yet progress in translating this priority into intervention development remains limited [13].

Concurrently, digital and technology‑enabled approaches are expanding rapidly in stroke rehabilitation, encompassing diverse technological modalities: video conference‑based telerehabilitation [14,15]; wearable sensors or activity trackers for remote monitoring [16,17]; virtual reality (VR) and augmented reality (AR) systems for immersive rehabilitation [18,19]; mobile health applications for self‑management [13,20]; artificial intelligence and machine learning for outcome prediction and personalized interventions [21,22]; robot‑assisted therapy [23,24]; exergames [25,26]; and brain‑computer interfaces with neurofeedback [27]. However, existing reviews have systematic limitations: digital health reviews focusing on stroke rehabilitation have primarily addressed motor outcomes and activities of daily living, without systematically considering fatigue as an independent outcome domain [28–30]; intervention reviews on PSF have concentrated on pharmacological approaches [31] or specific neuromodulation techniques [32]; and digital fatigue intervention reviews for other neurological conditions cannot be directly generalized due to the unique neurobiological mechanisms of PSF [33]. To our knowledge, no scoping review has systematically mapped the full range of technology categories and study designs for digital and technology‑enabled approaches targeting PSF.

A scoping review methodology was chosen over a systematic review because the literature on digital approaches for PSF is expected to be highly heterogeneous in study design, approach type, outcome measurement, and reporting quality, precluding meaningful meta‑analysis [34,35]. Unlike a focused systematic review on intervention effectiveness, a scoping review is specifically designed to map the breadth of available evidence, identify knowledge gaps, and clarify key concepts — precisely the objectives appropriate for the current stage of evidence development [36]. The resulting evidence map may help inform the design of future focused systematic reviews and guide priorities for primary research. This scoping review aims to systematically map the landscape of digital and technology‑enabled approaches for PSF. Specific objectives are: (1) to develop evidence maps to identify and categorize the types, purposes (assessment, monitoring, prevention, and treatment), and characteristics of existing digital and technology‑enabled approaches for PSF; (2) to describe the study designs, populations, settings, and outcome measures used in this body of literature; and (3) to identify evidence gaps and inform directions for future research.

## 2. Methods

### 2.1. Protocol design and registration

This scoping review will follow the Joanna Briggs Institute (JBI) methodology for scoping reviews, based on the methodological guidance updated by Peters et al.[36] in the JBI Manual for Evidence Synthesis, and on the foundational framework established by Arksey and O’Malley [37] and advanced by Levac et al.[38]. This protocol is reported in accordance with the PRISMA‑P 2015 statement [39] and the PRISMA‑ScR checklist [40] (see S1 File). The protocol has been registered on the Open Science Framework (OSF) to maximize transparency and facilitate independent verification (Registration https://doi.org/10.17605/OSF.IO/4WMEK). The study is currently in the protocol phase, and no database search or data extraction has been performed at the time of submission. Searches will commence once the protocol is finalized, and the review is expected to be completed by May 2027. Any protocol amendments will be documented in the OSF registration. Ethical approval is not required as this review does not involve primary data collection or human participants.

### 2.2. Eligibility criteria

Eligibility criteria are defined according to the JBI Population, Concept, Context (PCC) framework [36] to ensure clarity and reproducibility of the scoping boundaries.

Population: Adults (aged ≥18 years) with a clinical diagnosis of stroke (ischemic or hemorrhagic). Studies targeting PSF assessment, monitoring, or treatment must enroll participants with PSF; studies targeting PSF prevention may enroll stroke survivors regardless of baseline fatigue status. PSF is operationally defined as feelings of exhaustion, lack of energy, or weariness that begin after stroke, occur at any time from the acute phase onward, and are not fully explained by physical exertion, mood disorders, sleep disorders, or other medical conditions [41]. Fatigue attributable to a primary condition other than stroke (e.g., cancer‑related fatigue, multiple sclerosis fatigue) will be excluded. Fatigue may be established using a validated instrument (e.g., Fatigue Severity Scale [FSS], Checklist Individual Strength [CIS], Fatigue Assessment Scale [FAS], Multidimensional Fatigue Inventory [MFI], Neurological Fatigue Index for Stroke [NFI‑Stroke], Chalder Fatigue Scale, PROMIS Fatigue), structured clinical criteria (e.g., ICD‑11), or unstructured self‑report; the confirmation method is not an eligibility gate. Instead, the operational definition, instrument, cut‑off (e.g., FSS ≥ 4, FAS ≥ 22, CIS fatigue severity ≥35), fatigue domain (physical, cognitive, or mixed), and whether depression, sleep disorders, or other competing causes were assessed will be recorded for each study and used to stratify the evidence map. Transient ischemic attack (TIA), which does not involve brain infarction, is excluded. For studies with mixed populations (e.g., stroke and traumatic brain injury, or stroke and TIA), data will be included only if stroke‑specific subgroup data can be extracted separately.

Concept: Digital and technology‑enabled approaches whose core function relies on digital computation, data communication, human‑computer interaction, or programmable control, and whose purpose is to assess, monitor, prevent, or treat PSF. Approaches may be delivered alone or in combination with usual rehabilitation, provided that the digital/technology component is clearly described. Purely pharmacological approaches and conventional face‑to‑face rehabilitation without a described digital/technology component will be excluded. Each included approach will be coded by purpose (assessment, monitoring, prevention, or treatment; multiple purposes may apply) and by technology category using the pre‑specified classification scheme (S2 File), which will be piloted and revised during the pilot extraction phase.

Context: Any setting, including inpatient rehabilitation facilities, outpatient clinics, community settings, and home environments. No geographic restrictions apply.

Types of evidence source: This scoping review will include original research of any design (quantitative, qualitative, mixed‑methods), systematic reviews and scoping reviews, conference papers, dissertations, and registered trial protocols. Commentaries, editorials, and letters to the editor will be excluded unless they contain substantive descriptions of digital approaches for PSF. To prevent overlapping or uneven representation of the evidence, the following rules will be applied: (a) companion reports of the same study (e.g., protocol, main results, and secondary analyses) will be linked and treated as a single study unit, with the report containing the main empirical results designated as the primary record; (b) records reporting the same or overlapping study populations will be linked and counted once in the descriptive summaries; (c) primary studies already represented within an included systematic or scoping review will be linked to that review and will not be double‑counted in the numerical summaries; and (d) completed studies with empirical findings will be distinguished from ongoing studies, trial protocols, and conference abstracts without results, which will be presented separately in the evidence map.

### 2.3. Search strategy

The search strategy was first developed in consultation with a health sciences librarian and subsequently peer‑reviewed using the PRESS 2015 checklist [42]. A limited preliminary search of PubMed was conducted to identify relevant articles and refine search terms. The full strategy will then be adapted to each database’s syntax and controlled vocabulary. Eight electronic databases will be searched: PubMed, Embase, Web of Science, CINAHL, Cochrane CENTRAL, IEEE Xplore, ACM Digital Library, and PsycINFO. No time or study design filters will be applied to the database searches, but only English‑language records will be included. The search timeframe will be from database inception to the date of the search. In addition, reference lists of included studies and relevant reviews will be hand‑searched. Search results will be exported in RIS format and imported into NoteExpress for deduplication and screening. Gray literature sources: OpenGrey, ProQuest Dissertations and Theses Global, ClinicalTrials.gov, the World Health Organization International Clinical Trials Registry Platform (ICTRP), and Google Scholar. The complete search strategy for all databases is provided in S3 File; database searches will be rerun immediately before final analysis.

### 2.4. Study screening

All search results will be imported into NoteExpress for automatic deduplication. After deduplication, two reviewers will independently screen titles and abstracts. Records judged as potentially eligible by either reviewer will proceed to full‑text review. Two reviewers will independently assess the full‑text articles. Reasons for exclusion at the full‑text stage will be recorded and reported in the PRISMA‑ScR flow diagram. Disagreements will be resolved through consensus discussion; if consensus cannot be reached, a third reviewer will make a final decision. Before formal screening, a calibration exercise will be conducted on a random sample of 50 records, and Cohen’s kappa coefficient will be calculated for both the title/abstract and full‑text screening stages. For studies available only as conference abstracts or registered trials without published results, authors will be contacted twice at one‑week intervals via their institutional email addresses. If no response is received after two contact attempts, or if the authors explicitly indicate that additional data cannot be provided, the study will be included solely as a conference abstract or trial registration record and will be flagged in data extraction as “information incomplete”. The screening process will be documented using the PRISMA‑ScR flow diagram (see S4 File).

### 2.5. Data extraction

A standardized data extraction form will be developed in Excel and piloted on a random 10% sample of included studies (minimum five studies). The form will capture study characteristics, participant characteristics (including the operational definition of fatigue, the instrument and cut‑off used to establish fatigue, the fatigue domain, and whether competing causes were assessed), approach characteristics (including technology category, purpose, delivery mode, duration, frequency, and intensity), and context (S5 File). The purpose and technology category coding scheme (S2 File) will be tested during the pilot and revised if necessary. Two reviewers will independently extract data from each included study. Disagreements will be resolved by discussion or by a third reviewer.

### 2.6. Data analysis and presentations

Data will be synthesized using two complementary approaches, consistent with JBI guidance for scoping reviews [36]. Descriptive numerical summaries will characterize the included studies according to: year of publication and geographic distribution, country income classification, study design, participant characteristics, technology category, purpose, delivery mode, setting, and fatigue measurement method. Because the method used to establish fatigue is expected to vary, summaries will be stratified by confirmation method (validated instrument, structured clinical criteria, or unstructured self‑report), and completed studies with empirical findings will be presented separately from ongoing studies and records without results. Frequency analysis of technology categories and purposes will identify the distribution of evidence across the full spectrum. Inductive qualitative content analysis will supplement the quantitative mapping to identify themes and research gaps. This analysis will focus on key findings extracted from included studies, with particular attention to studies explicitly using qualitative or mixed‑methods designs. Data presentation will be structured around the review objectives, with evidence organized by technology category and purpose and aligned with the PCC framework elements.

### 2.7. Coding quality control

Before formal coding, the two reviewers will conduct a calibration exercise using a randomly selected 10% sample of studies that contain qualitative findings, to harmonize coding approaches and resolve ambiguities in the coding framework. During formal coding, the two reviewers will code independently; inter‑rater agreement will be calculated as a percentage agreement. An acceptable threshold of ≥80% agreement is set for this protocol. If agreement falls below this threshold, a third reviewer will adjudicate, and the coding framework will be recalibrated before re‑coding the discrepant items. Disagreements will first be resolved through iterative discussion; if no consensus is reached, a third reviewer will make a final decision. Consistent with scoping review methodology, no formal quality appraisal of individual studies will be performed. Findings will be reported according to PRISMA‑P [39] and PRISMA‑ScR [40].

## 3. Discussion

This protocol provides a rigorous and transparent methodological framework. Several methodological strengths support the credibility and reproducibility of this review. First, the search strategy covers eight databases spanning biomedicine, engineering, computer science, and psychology, as well as five gray literature sources — a breadth that exceeds most published PSF reviews [31,32]. Second, adherence to the JBI methodology for scoping reviews [36], supplemented by the PRISMA‑P [39] and PRISMA‑ScR [40] reporting guidelines, ensures that the conduct and reporting of the review conform to current international standards. Third, registration of the protocol on the OSF enhances transparency and reduces the risk of outcome reporting bias [43]. Fourth, the three‑module search strategy peer‑reviewed using PRESS 2015 [42] improves the reproducibility and sensitivity of the search. Fifth, the purpose‑by‑category coding scheme (with decision rules for overlapping categories) and the capture of fatigue confirmation methods as extraction variables provide clear and defensible scoping boundaries.

As a scoping review, this study will not include formal quality appraisal or meta‑analysis; this is consistent with the goal of evidence mapping and gap identification [36]. The field of digital health technology is evolving rapidly; including trial registries and gray literature sources helps mitigate the risk of overlooking newly developed technologies that have not yet been published. The planned content analysis of key findings is limited by the quality and completeness of reporting in the primary studies and serves gap identification rather than integrated qualitative evidence synthesis. Heterogeneity across studies in the definition and measurement of PSF [5] will itself be documented as a finding through the stratified presentation of results by fatigue confirmation method, contributing to methodological recommendations for standardization in the field.

The results of this scoping review will be disseminated through peer‑reviewed journal publication. The resulting evidence map will serve three purposes: (1) to provide clinicians and researchers with a comprehensive overview of available digital and technology‑enabled approaches for PSF, organized by technology category and purpose and mapped to fatigue dimensions; (2) to identify specific evidence gaps that warrant focused systematic reviews (e.g., in categories with sufficient evidence density such as telerehabilitation or VR interventions); and (3) to highlight under‑researched technology categories and PSF outcome domains, thereby informing funding priorities and trial designs.

## Supporting Information

S1 File PRISMA‑P and PRISMA‑ScR checklists. (DOCX)

S2 File Approaches classification scheme. (DOCX)

S3 File Complete search strategy. (DOCX)

S4 File PRISMA‑ScR flow diagram template. (DOCX)

S5 File Data extraction form. (DOCX)
