# Supplementary material for: Digital and technology‑enabled approaches for post‑stroke fatigue: A scoping review protocol
Source: PLoS One. 2026 Oct 29;21(10):e0352714. doi: 10.1371/journal.pone.0352714 (PMC13718960; doi:10.1371/journal.pone.0352714)
Supplement: Supplementary file 2 [file pone.0352714.s002.docx]

**Digital and Technology‑Enabled Approaches Classification Scheme**

Two independent dimensions are coded for every included record: **purpose** (Dimension 1) and **technology category** (Dimension 2). Decision rules for overlapping categories are listed below.

**Dimension 1: Purpose of the approach**

| **Code** | **Purpose** | **Definition** |
| --- | --- | --- |
| A | Assessment | The approach is used to measure, detect, or diagnose PSF (e.g., digital fatigue assessment tools, AI‑based screening). |
| B | Monitoring | The approach is used to track PSF over time (e.g., wearable‑based continuous monitoring, symptom diaries in apps). |
| C | Prevention | The approach aims to prevent or reduce the risk of PSF onset in stroke survivors without established fatigue. |
| D | Treatment | The approach aims to reduce established PSF severity or improve fatigue‑related outcomes. |

Multiple purposes may apply to a single approach and will all be coded.

**Dimension 2: Technology category**

| **Code** | **Category** | **Definition** | **Examples** |
| --- | --- | --- | --- |
| 1 | Mobile Health Applications (mHealth) | Smartphone/tablet apps for PSF self‑management, symptom tracking, or approach delivery. | iHealth app; fatigue tracker apps |
| 2 | Wearable Sensors and Activity Trackers | Inertial measurement units, accelerometers, smartwatches, and physiological sensors for continuous fatigue‑related monitoring. | Fitbit; Apple Watch; Garmin; ActiGraph |
| 3 | Virtual, Augmented, and Mixed Reality (VR/AR/MR) | Immersive and semi‑immersive systems for rehabilitation, exergaming, or fatigue assessment. | HTC Vive; Oculus; HoloLens |
| 4 | Telerehabilitation and Telehealth | Videoconferencing, remote monitoring platforms, and web‑based approach delivery systems. | Zoom‑based rehab; web portals |
| 5 | Artificial Intelligence and Machine Learning | Predictive models, decision support systems, natural language processing, and adaptive algorithms. | ML prediction models; chatbot approaches |
| 6 | Neuromodulation Technologies | Non‑invasive brain stimulation (tDCS, TMS, tACS) and related techniques when delivered with digital computation, programmable control, or real‑time neuro‑navigational software. | tDCS with neuro‑navigation; rTMS |
| 7 | Robotics and Exoskeletons | Robot‑assisted therapy devices, powered exoskeletons, and adaptive resistance systems. | Armeo; Lokomat; exoskeletons |
| 8 | Gaming and Exergaming | Serious games, gamified rehabilitation platforms, and commercial gaming systems (e.g., Wii, Kinect) applied to PSF. | Wii Fit; Kinect games; custom exergames |
| 9 | Brain‑Computer Interfaces and Neurofeedback | EEG‑based BCI, real‑time fMRI neurofeedback, and biofeedback systems. | EEG‑BCI rehab; HRV biofeedback |
| 10 | Multi‑modal | An approach reasonably classifiable into multiple categories, or whose main transmission mechanism remains unclear after discussion among the reviewers. | VR + telerehab; mHealth + wearable |
