# Supplementary material for: Digital and technology‑enabled approaches for post‑stroke fatigue: A scoping review protocol
Source: PLoS One. 2026 Oct 29;21(10):e0352714. doi: 10.1371/journal.pone.0352714 (PMC13718960; doi:10.1371/journal.pone.0352714)
Supplement: Supplementary file 3 [file pone.0352714.s003.docx]

# **Complete Search Strategies** **for Databases**

| **Database 1: PubMed** | |
| --- | --- |
| #1 | "Stroke"[MeSH] OR "Cerebrovascular Disorders"[MeSH] OR "Ischemic Stroke"[MeSH] OR "Hemorrhagic Stroke"[MeSH] OR "Intracranial Hemorrhages"[MeSH] OR stroke[tiab] OR post-stroke[tiab] OR poststroke[tiab] OR "cerebrovascular accident"[tiab] OR "cerebrovascular accidents"[tiab] OR CVA[tiab] OR "cerebral infarct"[tiab] OR "cerebral infarcts"[tiab] OR "intracerebral hemorrhage"[tiab] OR "intracerebral haemorrhage"[tiab] OR "intracranial hemorrhage"[tiab] OR "intracranial haemorrhage"[tiab] OR "subarachnoid hemorrhage"[tiab] OR "subarachnoid haemorrhage"[tiab] OR "brain ischemia"[tiab] OR "brain ischaemia"[tiab] |
| #2 | "Fatigue"[MeSH] OR "Fatigue Syndrome, Chronic"[MeSH] OR "Mental Fatigue"[MeSH] OR "Asthenia"[MeSH] OR fatigue[tiab] OR "post-stroke fatigue"[tiab] OR PSF[tiab] OR tiredness[tiab] OR exhaustion[tiab] OR lassitude[tiab] OR asthenia[tiab] OR weariness[tiab] OR "lack of energy"[tiab] OR "low energy"[tiab] OR fatigability[tiab] OR "energy conservation"[tiab] OR "energy management"[tiab] |
| #3 | "Mobile Applications"[MeSH] OR "Wearable Electronic Devices"[MeSH] OR "Fitness Trackers"[MeSH] OR "Virtual Reality"[MeSH] OR "Augmented Reality"[MeSH] OR "Telemedicine"[MeSH] OR "Telerehabilitation"[MeSH] OR "Artificial Intelligence"[MeSH] OR "Machine Learning"[MeSH] OR "Deep Learning"[MeSH] OR "Smartphone"[MeSH] OR "Robotics"[MeSH] OR "Exergaming"[MeSH] OR "Video Games"[MeSH] OR "Transcranial Magnetic Stimulation"[MeSH] OR "Transcranial Direct Current Stimulation"[MeSH] OR "Digital Technology"[MeSH] OR "Biomedical Technology"[MeSH] OR "Brain-Computer Interfaces"[MeSH] OR "Neurofeedback"[MeSH] OR digital[tiab] OR mHealth[tiab] OR m-health[tiab] OR eHealth[tiab] OR e-health[tiab] OR "mobile health"[tiab] OR app[tiab] OR apps[tiab] OR smartphone[tiab] OR smartphones[tiab] OR iPhone[tiab] OR Android[tiab] OR wearable[tiab] OR wearables[tiab] OR sensor[tiab] OR sensors[tiab] OR accelerometer[tiab] OR accelerometers[tiab] OR "activity tracker"[tiab] OR "activity trackers"[tiab] OR Fitbit[tiab] OR "Apple Watch"[tiab] OR Garmin[tiab] OR "virtual reality"[tiab] OR VR[tiab] OR "augmented reality"[tiab] OR AR[tiab] OR "mixed reality"[tiab] OR "head-mounted"[tiab] OR telerehabilitation[tiab] OR "tele-rehabilitation"[tiab] OR telehealth[tiab] OR telemedicine[tiab] OR "tele-health"[tiab] OR "tele-medicine"[tiab] OR "remote rehabilitation"[tiab] OR "remote monitoring"[tiab] OR videoconferencing[tiab] OR videoconference[tiab] OR "video-based"[tiab] OR "web-based"[tiab] OR "internet-based"[tiab] OR online[tiab] OR "internet-delivered"[tiab] OR computerized[tiab] OR computerised[tiab] OR "computer-based"[tiab] OR "computer-assisted"[tiab] OR "digital therapeutic"[tiab] OR "digital therapeutics"[tiab] OR DTx[tiab] OR gamification[tiab] OR gamified[tiab] OR "serious game"[tiab] OR "serious games"[tiab] OR exergame[tiab] OR exergames[tiab] OR "exer-game"[tiab] OR "active video game"[tiab] OR "active video games"[tiab] OR Wii[tiab] OR Kinect[tiab] OR robot[tiab] OR robots[tiab] OR robotic[tiab] OR "robot-assisted"[tiab] OR "robotic-assisted"[tiab] OR "artificial intelligence"[tiab] OR AI[tiab] OR "machine learning"[tiab] OR "deep learning"[tiab] OR "neural network"[tiab] OR "neural networks"[tiab] OR "natural language processing"[tiab] OR chatbot[tiab] OR chatbots[tiab] OR "conversational agent"[tiab] OR "conversational agents"[tiab] OR "brain-computer interface"[tiab] OR "brain computer interface"[tiab] OR BCI[tiab] OR neurofeedback[tiab] OR biofeedback[tiab] OR "transcranial magnetic"[tiab] OR TMS[tiab] OR "transcranial direct current"[tiab] OR tDCS[tiab] OR NIBS[tiab] OR "non-invasive brain stimulation"[tiab] |
| #4 | #1 AND #2 AND #3 |
| **Database 2: Embase (Ovid)** | |
| 1 | exp cerebrovascular disease/ OR exp stroke/ OR exp ischemic stroke/ OR exp brain hemorrhage/ |
| 2 | (stroke or post-stroke or poststroke or cerebrovascular accident* or CVA or cerebral infarct* or intracerebral hemorrhage* or intracerebral haemorrhage* or intracranial hemorrhage* or intracranial haemorrhage* or subarachnoid hemorrhage* or subarachnoid haemorrhage* or brain ischemia or brain ischaemia).ti,ab,kf. |
| 3 | 1 OR 2 |
| 4 | exp fatigue/ OR exp chronic fatigue syndrome/ OR exp mental fatigue/ OR exp asthenia/ |
| 5 | (fatigue or post-stroke fatigue or PSF or tiredness or exhaustion or lassitude or asthenia or weariness or lack of energy or low energy or fatigability or energy conservation or energy management).ti,ab,kf. |
| 6 | 4 OR 5 |
| 7 | exp mobile application/ OR exp wearable device/ OR exp fitness tracker/ OR exp virtual reality/ OR exp augmented reality/ OR exp telemedicine/ OR exp telerehabilitation/ OR exp artificial intelligence/ OR exp machine learning/ OR exp deep learning/ OR exp smartphone/ OR exp robotics/ OR exp exergaming/ OR exp video game/ OR exp transcranial magnetic stimulation/ OR exp transcranial direct current stimulation/ OR exp digital technology/ |
| 8 | (digital or mHealth or m-health or eHealth or e-health or mobile health or app or apps or smartphone* or iPhone or Android or wearable* or sensor* or accelerometer* or activity tracker* or Fitbit or Apple Watch or Garmin or virtual reality or VR or augmented reality or AR or mixed reality or head-mounted or telerehabilitation or tele-rehabilitation or telehealth or telemedicine or tele-health or tele-medicine or remote rehabilitation or remote monitoring or videoconferenc* or video-based or web-based or internet-based or online or internet-delivered or computerized or computerised or computer-based or computer-assisted or digital therapeutic* or DTx or gamif* or serious game* or exergame* or exer-game* or active video game* or Wii or Kinect or robot* or robot-assisted or robotic-assisted or artificial intelligence or AI or machine learning or deep learning or neural network* or natural language processing or chatbot* or conversational agent* or brain-computer interface* or brain computer interface* or BCI or neurofeedback or biofeedback or transcranial magnetic or TMS or transcranial direct current or tDCS or NIBS or non-invasive brain stimulation).ti,ab,kf. |
| 9 | 7 OR 8 |
| 10 | 3 AND 6 AND 9 |
| **Database 3: Web of Science Core Collection** | |
| #1 | TS=(stroke OR post-stroke OR poststroke OR "cerebrovascular accident" OR "cerebrovascular accidents" OR CVA OR "cerebral infarct" OR "cerebral infarcts" OR "intracerebral hemorrhage" OR "intracerebral haemorrhage" OR "intracranial hemorrhage" OR "intracranial haemorrhage" OR "subarachnoid hemorrhage" OR "subarachnoid haemorrhage" OR "brain ischemia" OR "brain ischaemia") |
| #2 | TS=(fatigue OR "post-stroke fatigue" OR PSF OR tiredness OR exhaustion OR lassitude OR asthenia OR weariness OR "lack of energy" OR "low energy" OR fatigability OR "energy conservation" OR "energy management") |
| #3 | TS=(digital OR mHealth OR m-health OR eHealth OR e-health OR "mobile health" OR app OR apps OR smartphone* OR iPhone OR Android OR wearable* OR sensor* OR accelerometer* OR "activity tracker" OR "activity trackers" OR Fitbit OR "Apple Watch" OR Garmin OR "virtual reality" OR VR OR "augmented reality" OR AR OR "mixed reality" OR "head-mounted" OR telerehabilitation OR "tele-rehabilitation" OR telehealth OR telemedicine OR "tele-health" OR "tele-medicine" OR "remote rehabilitation" OR "remote monitoring" OR videoconferenc* OR "video-based" OR "web-based" OR "internet-based" OR online OR "internet-delivered" OR computerized OR computerised OR "computer-based" OR "computer-assisted" OR "digital therapeutic" OR "digital therapeutics" OR DTx OR gamif* OR "serious game" OR "serious games" OR exergame* OR "exer-game" OR "exer-games" OR "active video game" OR "active video games" OR Wii OR Kinect OR robot* OR "robot-assisted" OR "robotic-assisted" OR "artificial intelligence" OR AI OR "machine learning" OR "deep learning" OR "neural network" OR "neural networks" OR "natural language processing" OR chatbot* OR "conversational agent" OR "conversational agents" OR "brain-computer interface" OR "brain computer interface" OR BCI OR neurofeedback OR biofeedback OR "transcranial magnetic" OR TMS OR "transcranial direct current" OR tDCS OR NIBS OR "non-invasive brain stimulation") |
| #4 | #1 AND #2 AND #3 |
| **Database 4: CINAHL (EBSCOhost)** | |
| S1 | (MH "Stroke+") OR (MH "Cerebrovascular Disorders+") OR (MH "Cerebral Ischemia+") OR (MH "Cerebral Hemorrhage+") OR TI(stroke OR post-stroke OR poststroke OR "cerebrovascular accident" OR "cerebrovascular accidents" OR CVA OR "cerebral infarct" OR "cerebral infarcts" OR "intracerebral hemorrhage" OR "intracerebral haemorrhage" OR "intracranial hemorrhage" OR "intracranial haemorrhage" OR "subarachnoid hemorrhage" OR "subarachnoid haemorrhage" OR "brain ischemia" OR "brain ischaemia") OR AB(stroke OR post-stroke OR poststroke OR "cerebrovascular accident" OR "cerebrovascular accidents" OR CVA OR "cerebral infarct" OR "cerebral infarcts" OR "intracerebral hemorrhage" OR "intracerebral haemorrhage" OR "intracranial hemorrhage" OR "intracranial haemorrhage" OR "subarachnoid hemorrhage" OR "subarachnoid haemorrhage" OR "brain ischemia" OR "brain ischaemia") |
| S2 | (MH "Fatigue+") OR (MH "Chronic Fatigue Syndrome+") OR (MH "Asthenia") OR TI(fatigue OR "post-stroke fatigue" OR PSF OR tiredness OR exhaustion OR lassitude OR asthenia OR weariness OR "lack of energy" OR "low energy" OR fatigability OR "energy conservation" OR "energy management") OR AB(fatigue OR "post-stroke fatigue" OR PSF OR tiredness OR exhaustion OR lassitude OR asthenia OR weariness OR "lack of energy" OR "low energy" OR fatigability OR "energy conservation" OR "energy management") |
| S3 | (MH "Mobile Applications+") OR (MH "Wearable Sensors+") OR (MH "Fitness Trackers") OR (MH "Virtual Reality+") OR (MH "Augmented Reality+") OR (MH "Telemedicine+") OR (MH "Telerehabilitation+") OR (MH "Artificial Intelligence+") OR (MH "Machine Learning+") OR (MH "Deep Learning") OR (MH "Smartphone+") OR (MH "Robotics+") OR (MH "Video Games+") OR (MH "Transcranial Magnetic Stimulation") OR (MH "Transcranial Direct Current Stimulation") OR (MH "Medical Technology+") OR TI(digital OR mHealth OR m-health OR eHealth OR e-health OR "mobile health" OR app OR apps OR smartphone* OR iPhone OR Android OR wearable* OR sensor* OR accelerometer* OR "activity tracker" OR "activity trackers" OR Fitbit OR "Apple Watch" OR Garmin OR "virtual reality" OR VR OR "augmented reality" OR AR OR "mixed reality" OR "head-mounted" OR telerehabilitation OR "tele-rehabilitation" OR telehealth OR telemedicine OR "tele-health" OR "tele-medicine" OR "remote rehabilitation" OR "remote monitoring" OR videoconferenc* OR "video-based" OR "web-based" OR "internet-based" OR online OR "internet-delivered" OR computerized OR computerised OR "computer-based" OR "computer-assisted" OR "digital therapeutic" OR "digital therapeutics" OR DTx OR gamif* OR "serious game" OR "serious games" OR exergame* OR "exer-game" OR "exer-games" OR "active video game" OR "active video games" OR Wii OR Kinect OR robot* OR "robot-assisted" OR "robotic-assisted" OR "artificial intelligence" OR AI OR "machine learning" OR "deep learning" OR "neural network" OR "neural networks" OR "natural language processing" OR chatbot* OR "conversational agent" OR "conversational agents" OR "brain-computer interface" OR "brain computer interface" OR BCI OR neurofeedback OR biofeedback OR "transcranial magnetic" OR TMS OR "transcranial direct current" OR tDCS OR NIBS OR "non-invasive brain stimulation") OR AB(digital OR mHealth OR m-health OR eHealth OR e-health OR "mobile health" OR app OR apps OR smartphone* OR iPhone OR Android OR wearable* OR sensor* OR accelerometer* OR "activity tracker" OR "activity trackers" OR Fitbit OR "Apple Watch" OR Garmin OR "virtual reality" OR VR OR "augmented reality" OR AR OR "mixed reality" OR "head-mounted" OR telerehabilitation OR "tele-rehabilitation" OR telehealth OR telemedicine OR "tele-health" OR "tele-medicine" OR "remote rehabilitation" OR "remote monitoring" OR videoconferenc* OR "video-based" OR "web-based" OR "internet-based" OR online OR "internet-delivered" OR computerized OR computerised OR "computer-based" OR "computer-assisted" OR "digital therapeutic" OR "digital therapeutics" OR DTx OR gamif* OR "serious game" OR "serious games" OR exergame* OR "exer-game" OR "exer-games" OR "active video game" OR "active video games" OR Wii OR Kinect OR robot* OR "robot-assisted" OR "robotic-assisted" OR "artificial intelligence" OR AI OR "machine learning" OR "deep learning" OR "neural network" OR "neural networks" OR "natural language processing" OR chatbot* OR "conversational agent" OR "conversational agents" OR "brain-computer interface" OR "brain computer interface" OR BCI OR neurofeedback OR biofeedback OR "transcranial magnetic" OR TMS OR "transcranial direct current" OR tDCS OR NIBS OR "non-invasive brain stimulation") |
| S4 | S1 AND S2 AND S3 |
| **Database 5: Cochrane CENTRAL** | |
| #1 | MeSH descriptor: [Stroke] explode all trees |
| #2 | MeSH descriptor: [Cerebrovascular Disorders] explode all trees |
| #3 | MeSH descriptor: [Ischemic Stroke] explode all trees |
| #4 | MeSH descriptor: [Hemorrhagic Stroke] explode all trees |
| #5 | MeSH descriptor: [Intracranial Hemorrhages] explode all trees |
| #6 | (stroke OR post-stroke OR poststroke OR "cerebrovascular accident" OR "cerebrovascular accidents" OR CVA OR "cerebral infarct" OR "cerebral infarcts" OR "intracerebral hemorrhage" OR "intracerebral haemorrhage" OR "intracranial hemorrhage" OR "intracranial haemorrhage" OR "subarachnoid hemorrhage" OR "subarachnoid haemorrhage" OR "brain ischemia" OR "brain ischaemia"):ti,ab,kw |
| #7 | #1 OR #2 OR #3 OR #4 OR #5 OR #6 |
| #8 | MeSH descriptor: [Fatigue] explode all trees |
| #9 | MeSH descriptor: [Fatigue Syndrome, Chronic] explode all trees |
| #10 | MeSH descriptor: [Mental Fatigue] explode all trees |
| #11 | MeSH descriptor: [Asthenia] explode all trees |
| #12 | (fatigue OR "post-stroke fatigue" OR PSF OR tiredness OR exhaustion OR lassitude OR asthenia OR weariness OR "lack of energy" OR "low energy" OR fatigability OR "energy conservation" OR "energy management"):ti,ab,kw |
| #13 | #8 OR #9 OR #10 OR #11 OR #12 |
| #14 | MeSH descriptor: [Mobile Applications] explode all trees |
| #15 | MeSH descriptor: [Wearable Electronic Devices] explode all trees |
| #16 | MeSH descriptor: [Virtual Reality] explode all trees |
| #17 | MeSH descriptor: [Augmented Reality] explode all trees |
| #18 | MeSH descriptor: [Telemedicine] explode all trees |
| #19 | MeSH descriptor: [Telerehabilitation] explode all trees |
| #20 | MeSH descriptor: [Artificial Intelligence] explode all trees |
| #21 | MeSH descriptor: [Machine Learning] explode all trees |
| #22 | MeSH descriptor: [Smartphone] explode all trees |
| #23 | MeSH descriptor: [Robotics] explode all trees |
| #24 | MeSH descriptor: [Video Games] explode all trees |
| #25 | MeSH descriptor: [Transcranial Magnetic Stimulation] explode all trees |
| #26 | MeSH descriptor: [Transcranial Direct Current Stimulation] explode all trees |
| #27 | (digital OR mHealth OR eHealth OR "mobile health" OR app OR apps OR smartphone* OR iPhone OR Android OR wearable* OR sensor* OR accelerometer* OR "activity tracker" OR Fitbit OR "Apple Watch" OR Garmin OR "virtual reality" OR VR OR "augmented reality" OR AR OR "mixed reality" OR telerehabilitation OR telehealth OR telemedicine OR "remote monitoring" OR videoconferenc* OR "web-based" OR "internet-based" OR online OR computerized OR "computer-based" OR "digital therapeutic" OR DTx OR gamif* OR "serious game" OR exergame* OR Wii OR Kinect OR robot* OR "artificial intelligence" OR AI OR "machine learning" OR "deep learning" OR "neural network" OR chatbot* OR "brain-computer interface" OR BCI OR neurofeedback OR biofeedback OR "transcranial magnetic" OR TMS OR "transcranial direct current" OR tDCS OR NIBS OR "non-invasive brain stimulation"):ti,ab,kw |
| #28 | #14 OR #15 OR #16 OR #17 OR #18 OR #19 OR #20 OR #21 OR #22 OR #23 OR #24 OR #25 OR #26 OR #27 |
| #29 | #7 AND #13 AND #28 |
| **Database 6: IEEE Xplore** | |
| ("All Metadata":stroke OR "All Metadata":post-stroke OR "All Metadata":poststroke OR "All Metadata":"cerebrovascular accident" OR "All Metadata":CVA OR "All Metadata":"cerebral infarct" OR "All Metadata":"intracerebral hemorrhage" OR "All Metadata":"intracranial hemorrhage" OR "All Metadata":"subarachnoid hemorrhage" OR "All Metadata":"brain ischemia")  AND  ("All Metadata":fatigue OR "All Metadata":"post-stroke fatigue" OR "All Metadata":PSF OR "All Metadata":tiredness OR "All Metadata":exhaustion OR "All Metadata":lassitude OR "All Metadata":asthenia OR "All Metadata":weariness OR "All Metadata":"lack of energy" OR "All Metadata":fatigability OR "All Metadata":"energy management")  AND  ("All Metadata":digital OR "All Metadata":mHealth OR "All Metadata":eHealth OR "All Metadata":"mobile health" OR "All Metadata":app OR "All Metadata":smartphone OR "All Metadata":wearable OR "All Metadata":sensor OR "All Metadata":accelerometer OR "All Metadata":"activity tracker" OR "All Metadata":Fitbit OR "All Metadata":"virtual reality" OR "All Metadata":VR OR "All Metadata":"augmented reality" OR "All Metadata":telerehabilitation OR "All Metadata":telehealth OR "All Metadata":telemedicine OR "All Metadata":"remote rehabilitation" OR "All Metadata":"remote monitoring" OR "All Metadata":"web-based" OR "All Metadata":"internet-based" OR "All Metadata":"computer-based" OR "All Metadata":gamif* OR "All Metadata":"serious game" OR "All Metadata":exergame OR "All Metadata":Wii OR "All Metadata":Kinect OR "All Metadata":robot* OR "All Metadata":"artificial intelligence" OR "All Metadata":AI OR "All Metadata":"machine learning" OR "All Metadata":"deep learning" OR "All Metadata":"neural network" OR "All Metadata":chatbot OR "All Metadata":"brain-computer interface" OR "All Metadata":BCI OR "All Metadata":neurofeedback OR "All Metadata":biofeedback OR "All Metadata":"transcranial magnetic" OR "All Metadata":TMS OR "All Metadata":"transcranial direct current" OR "All Metadata":tDCS OR "All Metadata":"non-invasive brain stimulation") | |
| **Database 7: ACM Digital Library** | |
| Title:(stroke OR post-stroke OR poststroke OR cerebrovascular OR CVA OR "cerebral infarct" OR "intracerebral hemorrhage" OR "brain ischemia") OR Abstract:(stroke OR post-stroke OR poststroke OR "cerebrovascular accident" OR CVA OR "cerebral infarct" OR "intracerebral hemorrhage" OR "brain ischemia") OR Keyword:(stroke OR rehabilitation OR neurorehabilitation)  AND  Title:(fatigue OR tiredness OR exhaustion OR "energy management") OR Abstract:(fatigue OR "post-stroke fatigue" OR PSF OR tiredness OR exhaustion OR lassitude OR asthenia OR weariness OR "lack of energy" OR fatigability OR "energy conservation" OR "energy management")  AND  Title:(digital OR mHealth OR eHealth OR mobile OR app OR smartphone OR wearable OR sensor OR "virtual reality" OR VR OR "augmented reality" OR telerehabilitation OR telehealth OR telemedicine OR "web-based" OR "computer-based" OR gamif* OR "serious game" OR exergame OR Wii OR Kinect OR robot* OR "artificial intelligence" OR AI OR "machine learning" OR "deep learning" OR chatbot* OR "brain-computer interface" OR BCI OR neurofeedback OR biofeedback) OR Abstract:(digital OR mHealth OR eHealth OR "mobile health" OR app OR apps OR smartphone* OR iPhone OR Android OR wearable* OR sensor* OR accelerometer* OR "activity tracker" OR Fitbit OR "Apple Watch" OR Garmin OR "virtual reality" OR VR OR "augmented reality" OR AR OR "mixed reality" OR telerehabilitation OR telehealth OR telemedicine OR "remote rehabilitation" OR "remote monitoring" OR videoconferenc* OR "web-based" OR "internet-based" OR online OR computerized OR "computer-based" OR "digital therapeutic" OR DTx OR gamif* OR "serious game" OR exergame* OR Wii OR Kinect OR robot* OR "artificial intelligence" OR AI OR "machine learning" OR "deep learning" OR "neural network" OR chatbot* OR "brain-computer interface" OR BCI OR neurofeedback OR biofeedback) | |
| **Database 8: PsycINFO (EBSCOhost)** | |
| S1 | (DE "Cerebrovascular Accidents" OR DE "Cerebral Hemorrhage" OR DE "Cerebral Ischemia") OR TI(stroke OR post-stroke OR poststroke OR "cerebrovascular accident" OR "cerebrovascular accidents" OR CVA OR "cerebral infarct" OR "cerebral infarcts" OR "intracerebral hemorrhage" OR "intracranial hemorrhage" OR "subarachnoid hemorrhage" OR "brain ischemia") OR AB(stroke OR post-stroke OR poststroke OR "cerebrovascular accident" OR "cerebrovascular accidents" OR CVA OR "cerebral infarct" OR "cerebral infarcts" OR "intracerebral hemorrhage" OR "intracranial hemorrhage" OR "subarachnoid hemorrhage" OR "brain ischemia") |
| S2 | (DE "Fatigue" OR DE "Chronic Fatigue Syndrome" OR DE "Asthenia") OR TI(fatigue OR "post-stroke fatigue" OR PSF OR tiredness OR exhaustion OR lassitude OR asthenia OR weariness OR "lack of energy" OR "low energy" OR fatigability OR "energy conservation" OR "energy management") OR AB(fatigue OR "post-stroke fatigue" OR PSF OR tiredness OR exhaustion OR lassitude OR asthenia OR weariness OR "lack of energy" OR "low energy" OR fatigability OR "energy conservation" OR "energy management") |
| S3 | (DE "Mobile Applications" OR DE "Virtual Reality" OR DE "Augmented Reality" OR DE "Telemedicine" OR DE "Artificial Intelligence" OR DE "Machine Learning" OR DE "Smartphones" OR DE "Robotics" OR DE "Computer Games" OR DE "Transcranial Magnetic Stimulation" OR DE "Wearable Devices" OR DE "Digital Interventions" OR DE "Electronic Health Services") OR TI(digital OR mHealth OR m-health OR eHealth OR e-health OR "mobile health" OR app OR apps OR smartphone* OR iPhone OR Android OR wearable* OR sensor* OR accelerometer* OR "activity tracker" OR Fitbit OR "Apple Watch" OR Garmin OR "virtual reality" OR VR OR "augmented reality" OR AR OR "mixed reality" OR telerehabilitation OR telehealth OR telemedicine OR "remote rehabilitation" OR "remote monitoring" OR videoconferenc* OR "web-based" OR "internet-based" OR online OR "internet-delivered" OR computerized OR computerised OR "computer-based" OR "computer-assisted" OR "digital therapeutic" OR DTx OR gamif* OR "serious game" OR "serious games" OR exergame* OR Wii OR Kinect OR robot* OR "artificial intelligence" OR AI OR "machine learning" OR "deep learning" OR "neural network" OR chatbot* OR "brain-computer interface" OR BCI OR neurofeedback OR biofeedback OR "transcranial magnetic" OR TMS OR "transcranial direct current" OR tDCS OR NIBS OR "non-invasive brain stimulation") OR AB(digital OR mHealth OR m-health OR eHealth OR e-health OR "mobile health" OR app OR apps OR smartphone* OR iPhone OR Android OR wearable* OR sensor* OR accelerometer* OR "activity tracker" OR Fitbit OR "Apple Watch" OR Garmin OR "virtual reality" OR VR OR "augmented reality" OR AR OR "mixed reality" OR telerehabilitation OR telehealth OR telemedicine OR "remote rehabilitation" OR "remote monitoring" OR videoconferenc* OR "web-based" OR "internet-based" OR online OR "internet-delivered" OR computerized OR computerised OR "computer-based" OR "computer-assisted" OR "digital therapeutic" OR DTx OR gamif* OR "serious game" OR "serious games" OR exergame* OR Wii OR Kinect OR robot* OR "artificial intelligence" OR AI OR "machine learning" OR "deep learning" OR "neural network" OR chatbot* OR "brain-computer interface" OR BCI OR neurofeedback OR biofeedback OR "transcranial magnetic" OR TMS OR "transcranial direct current" OR tDCS OR NIBS OR "non-invasive brain stimulation") |
| S4 | S1 AND S2 AND S3 |

## **Gray Literature Sources**

|  | **URL** | **Search string** |
| --- | --- | --- |
| OpenGrey | http://www.opengrey.eu/ | (stroke OR post-stroke OR cerebrovascular) AND (fatigue OR tiredness OR exhaustion) AND (digital OR mobile OR app OR virtual reality OR telemedicine OR wearable OR sensor) |
| ProQuest Dissertations and Theses Global | https://www.proquest.com | (stroke OR post-stroke OR cerebrovascular) AND (fatigue OR "post-stroke fatigue") AND (digital OR mobile OR app OR "virtual reality" OR telehealth OR wearable OR sensor)  Filter: All dates; Source type → Doctoral Dissertations + Master's Theses |
| ClinicalTrials.gov | https://clinicaltrials.gov/ | Condition/Disease: stroke Other terms: fatigue digital mobile app virtual reality wearable telehealth Status: All studies (including recruiting, completed, terminated) |
| World Health Organization International Clinical Trials Registry Platform | https://trialsearch.who.int/ | stroke AND fatigue AND (digital OR mobile OR app OR virtual reality OR telehealth OR wearable) |
| Google Scholar | https://scholar.google.com/ | ("post-stroke fatigue" OR "stroke fatigue") AND (digital OR mobile OR app OR "virtual reality" OR telehealth OR wearable OR sensor)  Settings: Sort by relevance; screen first 200 results; incognito/private browsing mode |
