# Supplementary material for: Digital and technology‑enabled approaches for post‑stroke fatigue: A scoping review protocol
Source: PLoS One. 2026 Oct 29;21(10):e0352714. doi: 10.1371/journal.pone.0352714 (PMC13718960; doi:10.1371/journal.pone.0352714)
Supplement: Supplementary file 5 [file pone.0352714.s005.docx]

**Draft Data Extraction Form**

| **Section** | **Field** | **Notes** |
| --- | --- | --- |
| **Study characteristics** | Authors |  |
|  | Year of publication |  |
|  | Country |  |
|  | Study design |  |
|  | Sample size |  |
|  | Funding source*1 |  |
|  | Study status | Completed with empirical results / ongoing / trial protocol / conference abstract without results |
|  | Companion report / duplicate population | Link to primary record if this record is a companion report or shares a study population with another record |
| **Participant characteristics** | Age |  |
|  | Sex distribution |  |
|  | Stroke type*2 | Ischemic, hemorrhagic, mixed |
|  | Time post‑stroke*3 | Acute (<1 month), subacute (1–6 months), chronic (>6 months), mixed/unspecified |
|  | Fatigue operational definition | Definition of fatigue used by the study, verbatim or paraphrased |
|  | Fatigue confirmation method | Validated instrument / structured clinical criteria / unstructured self‑report |
|  | Instrument and cut‑off | e.g., FSS ≥4, FAS ≥22, CIS fatigue severity ≥35 |
|  | Fatigue domain | Physical / cognitive / mixed / not reported |
|  | Competing causes assessed | Whether depression, sleep disorders, or other competing causes were assessed and how (yes/no/not reported) |
|  | Stroke severity*4 | NIHSS or equivalent, where reported |
|  | Secondary outcomes*5 |  |
| **Approach characteristics** | Purpose*6 | Assessment / monitoring / prevention / treatment (multiple allowed) |
|  | Technology category coding | Per S2 File, Dimension 2 |
|  | Specific technology description |  |
|  | Delivery mode*7 | In‑person, remote, hybrid |
|  | Duration |  |
|  | Frequency |  |
|  | Intensity |  |
|  | Comparator or control condition |  |
|  | Adverse events |  |
| **Context** | Setting*8 | Inpatient, outpatient, community, home |
|  | Country |  |
|  | Income classification*9 | Low, lower‑middle, middle, upper‑middle, high |

*1: Funding source categorized as: Academic/government, non‑profit/philanthropic, industry, mixed, or not reported

*2: Stroke type: ischemic, hemorrhagic, mixed (TIA‑only populations are excluded)

*3: Time post‑stroke categorized as: acute (<1 month), subacute (1–6 months), chronic (>6 months), mixed/unspecified (study includes multiple phases or does not specify)

*4: Stroke severity: NIHSS or equivalent, where reported

*5: Secondary outcomes reported: Quality of life, mood/depression, anxiety, physical function/mobility, activities of daily living, participation, sleep, cognitive function, self‑efficacy, caregiver burden, cost‑effectiveness, other (specify)

*6: Purpose coded per S2 File Dimension 1; multiple purposes may apply

*7: Delivery mode: in‑person, remote, hybrid

*8: Setting: inpatient, outpatient, community, home

*9: Income classification: low, lower‑middle, middle, upper‑middle, high
